# Establishing a community-based participatory research partnership among people who use drugs in Ottawa: the PROUD cohort study

**DOI:** 10.1186/1477-7517-11-26

**Published:** 2014-10-13

**Authors:** Lisa Lazarus, Ashley Shaw, Sean LeBlanc, Alana Martin, Zack Marshall, Kristen Weersink, Dolly Lin, Kira Mandryk, Mark W Tyndall, the PROUD Community Advisory Committee

**Affiliations:** Ottawa Hospital Research Institute, 216 Murray Street, Ottawa, ON K1N 5N1 Canada; Drug Users Advocacy League, Ottawa, Canada; Division of Community Health and Humanities, Memorial University of Newfoundland, St John’s, Canada; Department of Medicine, University of Ottawa, Ottawa, Canada; Ottawa Public Health, Ottawa, Canada; Division of Infectious Diseases, University of Ottawa at the Ottawa Hospital, Ottawa, Canada

**Keywords:** CBPR, Drug use, Harm reduction, HIV, Ottawa

## Abstract

**Background:**

Grounded in a community-based participatory research (CBPR) framework, the PROUD (Participatory Research in Ottawa: Understanding Drugs) Study aims to better understand HIV risk and prevalence among people who use drugs in Ottawa, Ontario. The purpose of this paper is to describe the establishment of the PROUD research partnership.

**Methods:**

PROUD relies on peers’ expertise stemming from their lived experience with drug use to guide all aspects of this CBPR project. A Community Advisory Committee (CAC), comprised of eight people with lived experience, three allies and three ex-officio members, has been meeting since May 2012 to oversee all aspects of the project. Eleven medical students from the University of Ottawa were recruited to work alongside the committee. Training was provided on CBPR; HIV and harm reduction; and administering HIV point-of-care (POC) tests so that the CAC can play a key role in research design, data collection, analysis, and knowledge translation activities.

**Results:**

From March-December 2013, the study enrolled 858 participants who use drugs (defined as anyone who has injected or smoked drugs other than marijuana in the last 12 months) into a prospective cohort study. Participants completed a one-time questionnaire administered by a trained peer or medical student, who then administered an HIV POC test. Recruitment, interviews and testing occurred in both the fixed research site and various community settings across Ottawa. With consent, prospective follow-up will occur through linkages to health care records available through the Institute for Clinical and Evaluation Sciences.

**Conclusion:**

The PROUD Study meaningfully engaged the communities of people who use drugs in Ottawa through the formation of the CAC, the training of peers as community-based researchers, and integrated KTE throughout the research project. This project successfully supported skill development across the team and empowered people with drug use experience to take on leadership roles, ensuring that this research process will promote change at the local level. The CBPR methods developed in this study provide important insights for future research projects with people who use drugs in other settings.

## Background

Community-based participatory research (CBPR) is an approach that prioritizes community inclusion throughout the research process, with community members bringing their knowledge and lived experience to the research with the goal of combining education and action to achieve social change that would benefit the community [1–3]. There has been a growing recognition of the need to more meaningfully involve community members and organizational partners, alongside academic researchers, in public health research [4]. Peer-led drug user advocacy groups have themselves been calling for more meaningful involvement through the promotion of the practice “Nothing For Us, Without Us” [5].

Although there have been efforts to more meaningfully engage communities in research, peers are rarely involved in every stage of the development, design, and dissemination of research projects [6, 7]. This is especially true for projects involving people who use drugs, where few projects have engaged peer researchers in roles that go beyond consultation and recruitment [8, 9]. Most research engaging people with lived experience with drug use has focused on collaborating with individuals who no longer consume drugs and who may no longer represent consumer perspectives [8, 10, 11]. People who still consume drugs may not be given the same opportunities to participate, as researchers may fear how their drug use could impact both the individual’s “recovery process” and project outcomes [8]. Furthermore, peers often do not receive the support necessary to fully engage in the work [10].

The geographic distribution of drug users in Ottawa, as well as structural and cultural factors, have been implicated in perpetuating high rates of HIV infection [12–14] and have frustrated prevention efforts [15]. As the rate of HIV infection in Ottawa remains high, it is imperative to work with the communities of people who use drugs in order to better understand the “risk environments” [16] that lead to an increased burden of HIV infection and barriers to accessing prevention, testing and treatment. People who use drugs can play a critical role as they are best able to identify the needs of a community that outsiders often know little about.

The PROUD (Participatory Research in Ottawa: Understanding Drugs) Study aims to better understand the HIV risk environment and prevalence of HIV among people who use drugs in Ottawa, Ontario. This study relies on peers’ expertise stemming from their lived experience to guide all aspects of the project. From the planning stages of this proposal through to implementation and action for community change, we ground our process in a community-based participatory research approach. In addition, PROUD has integrated CBPR principles with large-scale, quantitative cohort methods that draw on the Rhodes ‘Risk Environment Framework’ for understanding and reducing drug-related harms [16]. The Rhodes model posits that individual risk behaviours, health inequities, and differential access to health services are inextricably linked to the socio-structural contexts that shape daily lived experiences. In recognizing that community members themselves are experts in the environments of risk that they experience, we operationalize the Risk Environment Framework through CBPR processes. This paper will outline the development and methods of the PROUD study, as well as highlight strengths, challenges, and ethical concerns that present themselves in CBPR.

## Methods

### Community-based participatory research - planning and development of the PROUD study

#### Forming the community advisory committee

In partnership with the Drug User Advocacy League (DUAL), a Community Information Session was held in March 2012. In response to a call for Community Advisory Committee members, individuals who self-identified as having lived experience with drug use and/or stakeholders completed an application form describing their experiences with drug use and their interest in the project. Applicants were interviewed by both a peer and an academic researcher. Twenty-one applicants were interviewed, and fourteen were selected to join the Community Advisory Committee (CAC), including eight people with lived experience with drug use, three allied frontline support workers, and three ex-officio representatives from organizations working to improve the health and rights of Ottawa’s drug using communities. Gender, ethnicity, age, drug use, HIV status, experience with sex work, sexual identity, and francophone and Aboriginal perspectives were taken into consideration when selecting CAC members to ensure that the lived experiences of people facing multiple and intersecting oppressions were represented.

The CAC has been meeting since May 2012. This group was brought together to contribute to the research design, data collection, analysis, and knowledge translation stages of the PROUD Study and will continue to meet throughout all phases of the research process. CAC members receive an honorarium for their work on the project ($25/meeting), recognizing their role as expert consultants [17, 18]. CAC meetings are facilitated by members with lived experience, alongside the academic researchers and study coordinators, to ensure collaboration, transparent decision-making, and meaningful CAC control over the research process. A different CAC member is asked to volunteer to facilitate each meeting. The facilitator meets with one of the two study coordinators ahead of time to review the agenda and plan for the meeting. The principal investigator also attends all CAC meetings.

### Survey development

The first major activity of the CAC was to develop the survey tool. The CAC worked together to identify research themes that they deemed relevant to understanding the HIV risk environment in their communities. Taking a ground up approach, the CAC wrote down themes and subthemes on boards positioned around the meeting room. Subthemes were then merged, and eight main themes were agreed upon including: characteristics of drug users in Ottawa; drug use patterns; access to harm reduction services; health status and health care access; sexual activities and history; connections to community; housing and homelessness; and the influence of law enforcement. Working groups of 2–3 members developed survey questions within the themes that most interested them. Research coordinators edited questions to fit a survey format. Survey drafts were shared with the CAC, who provided input on wording, question order, and the length of the survey. Group debate and, when possible, consensus was sought in decision-making about which questions to include in the final tool. Questions were chosen based on usefulness and appropriateness for the target community, and on feedback from the broader community obtained during piloting.

The CAC members, along with medical students, piloted the survey with 26 participants to determine cultural appropriateness, internal consistency, construct validity, and the ideal survey length. CAC members were each asked to recruit two members from their contacts who were also members of the target population to pilot the survey. A brief feedback form was completed by both the interviewer and participant that identified confusing or challenging questions. Following the piloting, the CAC met again to integrate findings from the piloting and finalize the survey. The piloting process also provided peers and students with the opportunity to practice interviewing and address any questions or concerns about the interview process ahead of study initiation.

### Co-learning opportunities with medical students

As with CAC recruitment, an information night was held for University of Ottawa medical students in September 2012. Recruitment of medical students was restricted to students in their second year of studies as their schedules were most compatible with the project’s timelines. Twenty-three students were interviewed by both a peer and academic researcher, and eleven were selected to work alongside the advisory committee based on their past work experience in community-based settings and their interest in inner city health. Although opportunities remain infrequent for medical students to engage in community-based research, there have been a few successful examples of medical student involvement in CBPR [19–22]. Medical student participation alongside peers provided avenues for co-learning and exposed students to inner-city health issues early in their careers [20–22]. The nature of these teams also worked to challenge traditional power imbalances between health professionals and drug users by providing opportunities to work together as peers [3, 23].

### Building capacity - training of the research team

The CAC and medical student interviewers came together for a week of skill building in September 2012. Training was provided on CBPR; HIV and harm reduction; the roles of peer and medical student interviewers; research ethics; and interviewing skills. Training on administering HIV point-of-care (POC) tests was provided by Ottawa Public Health. The training modules were both specifically developed for the PROUD study and also drew on established trainings in research ethics [24] and peer research [25]. Training in research skills included special attention to confidentiality, privacy, informed consent, and the Tri-Council Policy Statement 2; verbal and non-verbal communication; diversity of the study participants; and administering quantitative surveys [26, 27]. The purpose of the training was two-fold. First and foremost, the training served to prepare the research team to take on more active roles in data collection, as CAC members and medical students also served as recruiters, interviewers and HIV testers. Second and equally importantly, the training brought together the large group of peer and medical student researchers in order to foster relationships and build trust among the team prior to the launch of the project. Further training will be provided on data analysis and KTE to ensure that community members remain meaningfully involved in all stages of the project.

### Participant recruitment

Enrolment into the study took place between March and December 2013. All participants were 16 years or older, had injected or smoked drugs other than marijuana in the past 12 months, and had lived in Ottawa for at least three months at the time of enrolment. Maps indicating the geographic concentration of discarded needles and the frequency of harm reduction service use were used to identify key recruitment areas. Notably, City Ward 12, home to Rideau-Vanier, was the location of 80% of all discarded needles found by Ottawa Public Health in 2013 [28]. Hence, the first wave of recruitment was conducted at the research space located in Ottawa’s downtown ByWard Market, in the heart of City Ward 12. Other priority neighbourhoods were determined based on the expert knowledge of the CAC who are familiar with relevant social networks combined with discussions with organizations that serve people who use drugs. Interview spaces were secured in other priority neighbourhoods through in-kind support from partner agencies. Although probability sampling was not feasible in this population [29], the composition of the CAC made it ideally positioned to employ a targeted recruitment approach to ensure that the lived experiences of people facing multiple and intersecting oppressions were represented among the study participants. This targeted recruitment approach sampled participants from various locations, at various times of day, and from multiple, diverse social networks.

In pairs, PROUD researchers recruited participants during defined shifts. Recruiters wearing identifying lanyards approached potential participants in a variety of street-based and public locations, presented study information cards, followed a verbal recruitment script to determine interest and eligibility, and scheduled interview times. Interviews were scheduled on the same day that participants were recruited in order to minimize the number of missed appointments. Recruitment also took place in the neighbourhoods where the interview was to occur to ensure that participants did not have to travel far distances. Scheduling appointments in advance served to reduce the potential for long wait-times and crowd control issues. Participation in the PROUD study included a one-time, interviewer administered, iPad-based quantitative survey, an HIV POC test, and prospective follow-up through health record linkages available through the Institute for Clinical and Evaluative Sciences (ICES) in Ontario. Consent was obtained for each element of the study and participants could choose to opt-in to the HIV POC test and/or data linkages. All study documents were translated into French and French-speaking interviewers were available to administer the survey and HIV POC testing. Consistent with other cohort research projects, a cash honorarium (CAN $20.00) was offered to participants after completing the survey portion of the study, regardless of whether participants choose to opt-in to the HIV POC test and/or data linkages. Participation in the study took approximately 1.5 hours. This research has received ethical approval from the Ottawa Hospital and Ottawa Public Health Research Ethics Boards.

### Interview data

The questionnaire includes individual-level variables, including socio-demographic information (age, gender, income, neighbourhood) and drug use (age at first use, frequency of type-specific drug use, modes and locations of drug use, circumstances and frequency of overdose); interpersonal variables, including sexual history (number of regular, casual and client sex partners, condom use and drug use with partner type), and connection to community (access to social and peer support services, social networks, history of suicidal ideation/attempts); and environmental-structural variables, such as harm reduction (access to new needles and pipes, equipment sharing practices), housing and homelessness (housing stability, evictions, access to housing supports, satisfaction with housing, drug use within residences), legal matters (red zones, incarceration) and health (access to health services, self-reported mental health, HIV and HCV diagnoses, knowledge of HIV testing and treatment, and ARV adherence). Data was collected using iPads that contained an electronic version of the survey. The instrument was specially designed to enter responses using large button icons. The data collection was web-based with no information stored on the iPad itself. All data is stored in a secure server housed at the Methods Centre at the Ottawa Hospital.

### HIV point-of-care testing and referral

Following the survey, participants were offered a nominal HIV POC test (bioLytical INSTI test), administered by a peer or medical student researcher. Both groups underwent rigorous training on the administration of HIV POC tests, along with training in pre- and post-test counselling, and were certified through Ottawa Public Health (OPH) prior to administering the tests. Through partnership with OPH, a nurse also provided on-site back up for result interpretation, pre- and post-test counselling and was available for onsite serology, referral, and quality assurance of the HIV POC tests and testing process.

### Data linkages and prospective follow-up

Prospective follow-up will be conducted for participants who consented to health-related database linkages within Ontario through the Institute for Clinical and Evaluative Sciences (ICES). Identifying information (name, date of birth, postal code) collected at the time of recruitment will be submitted to ICES and all analyses will be performed within the ICES centre. Based on past ICES linkage projects with other marginalized groups, it is anticipated that 90-95% of cohort participants will be successfully linked. The results of the linkages will be returned in tabular form with no ability to identify individuals in the cohort. This collection of linked databases will provide important information on access to community and hospital-based health care services; drug benefits claims; diagnostic and mortality data; and Public Health data to assess outcomes for people living with HIV, including HIV testing history, CD4, and viral load counts.

## Results and discussion

CBPR frameworks have traditionally been reserved for smaller-scale qualitative and ethnographic research projects. The PROUD Study uniquely applies CBPR principles to engage the communities of people who use drugs in the development of a large-scale prospective cohort study. Along with developing new models of CBPR among people who use drugs, the PROUD Study will also advance knowledge of the epidemiology of HIV among people who use drugs in the Ottawa area and develop novel community-based strategies for HIV testing. From March to December 2013, the PROUD study recruited 858 people with drug use experience into the PROUD cohort. 593 (69.1%) participants were recruited from Ottawa’s downtown core, where the majority of drug use is concentrated. A further 265 (30.9%) participants have been recruited from other known areas within Ottawa where people who use drugs are more typically located. The median age of participants was 43 years [age range of 17–70 years]. Nearly three-quarters of the participants (*n* = 638, 74.4%) were male and 156 (18.2%) identified as Aboriginal. Over two-thirds of participants (*n* = 589, 68.6%) had ever injected drugs and almost half (*n* = 409, 47.7%) had injected in the past year. Sixty-one participants (7.1%) self-reported being HIV+, with the HIV prevalence rising to 11.0% (*n* = 45/409) among those participants who had injected in the past year. Nearly 80% of participants who were offered HIV POC testing agreed to a test and over 90% of participants consented to health-related database linkages. Detailed findings of the survey analysis, HIV POC testing results and ICES linkages will be presented in future manuscripts.

### CBPR model for research with people who use drugs

The PROUD Study has demonstrated that CBPR can be successfully applied to the development and implementation of large-scale cohort studies. Central to developing strong CBPR models of research is community engagement, trust, collaboration and ownership [30, 31]. From the beginning, PROUD partnered with the local Drug User Advocacy League (DUAL) to form a community advisory committee (CAC) which would oversee all aspects of the PROUD research project. The chairperson of DUAL is Co-Principal Investigator of PROUD. By partnering with DUAL and forming strong collaborations with other established community organizations, PROUD built on existing relationships that these organizations hold with the communities of people who use drugs in Ottawa to recruit members into the CAC. The establishment of the CAC provided an excellent platform to answer important research questions, while creating opportunities for community members to engage with the project and to co-develop the partnership and its work.

Although CBPR provides opportunities for community collaboration, it is not without its challenges. In CBPR, it can be difficult to develop fully equitable partnerships between academic and community researchers [4, 32, 33]. The key to continued CAC participation was to establish trust in the process through transparent approaches to communication and an honest acknowledgement of power differences between academic and peer researchers [4]. By providing meaningful leadership roles to CAC members, characterized by regular consultation and collaborative approaches to decision-making and resource use [34], we were able to foster co-ownership that supported long-term engagement [35]. These collaborative process considerations were elaborated in the CAC Terms of Reference, which were adapted from the Trans PULSE study [32] and agreed upon by all members. Establishing decision-making processes was important to mitigate the challenge of conflicting ideas, values, and priorities within the CAC and the broader community [36].

Another consideration in CBPR is the burden of time and resources required of CAC members to participate as equal partners. One method of mitigating this burden is by providing paid opportunities. The difficult balance between respectful and adequate compensation for work done and the potential for coercion to participate were carefully considered when developing honorarium protocols [17]. Although all CAC members expressed interest in participating in the project, it proved difficult for some individuals to commit the time needed to engage in the CBPR process. Consideration was made to avoid overburdening community members with unnecessary tasks or meetings, and effort was made to allow CAC members to set the pace of the project. Some members were able to stay committed to the project by scaling back their involvement at certain points in time, or tailoring roles to best fit their individual strengths. As in other studies, short working shifts were set as to not overly tax peer researchers [9]. When CAC members decided to step back from the committee, a process was developed to include new members to retain high levels of community participation.

Finally, the formation of a CAC itself could spark conflict between community members [34]. Defining community can be challenging, as there is no such thing as a homogeneous group of drug users. Community members often have differing ideas about community needs and values. Challenges arose related to pre-existing relationships, causing tensions to occasionally flare between CAC members. Conflicts were often effectively deescalated through mediation, compromise, support and a mutual agreement on the importance of the project. We recognize that when working with peer researchers with lived experience with drug use, it is important to remain mindful of potential ethical concerns and emotional impacts. These topics were explored throughout the CAC training, in collaboration with the Learning Exchange for HIV/AIDS Peer Researchers’ initiative [25]. We have also incorporated recommendations from Flicker, Roche, & Guta [37] to ensure that appropriate support and supervision were in place and implemented strategies for ongoing debriefing as part of our practice.

An important component of the PROUD research partnership is a parallel evaluation of our community-based processes to better understand the experiences of the diverse team throughout all stages of the research project. All members of the research team, including CAC members, medical students, study coordinators and the lead researcher have opted-in to participating in in-depth interviews exploring their involvement with the project. The findings from this evaluation are expected to provide further insights into the benefits and challenges of CBPR partnerships with people who use drugs.

### Advancing epidemiological evidence in Ottawa

The PROUD study is the largest study of its kind in Ottawa and one of the largest cohorts of people who use drugs nationally. A major challenge of cohort studies with people who use drugs is enrolling participants [29]. Our street-based peer recruitment plan was designed specifically to engage those who may not have otherwise participated in the research [38]. As the interviews were conducted in-person, interviewers could support culturally sensitive, low-barrier participation despite potential literacy, physical, or cognitive challenges among participants. The visibility of peer researchers also fostered community ownership of the project, a step towards mitigating the challenge of researcher mistrust [39]. We are very cognizant of the potential for coercion among people with limited access to income even when small amounts of money are involved. Steps were put in place to fully inform potential participants and explain the consent in plain language. Recruiter and interviewer honorariums were given for each 4-hour shift and were not linked to participant recruitment quotas. This approach sought to minimize the risk of participant coercion [40]. People who were under the influence of substances had their enrolment deferred if concerns existed that their consent may be compromised.

There were also ethical issues related to privacy and confidentiality among research participants. As a general practice, CAC members decided not to interview participants that they knew well from outside of the study. Recruitment was monitored closely by the research coordinators to ensure that privacy was protected, referrals were made with full consent, and that there was no undue pressure to participate. By employing a peer-driven approach to enrolling a representative sample of drug users in Ottawa, the PROUD Study will advance knowledge of the epidemiology of HIV among drug users in the Ottawa area and will provide important information for public health decision makers to inform harm reduction programming.

### Novel models of peer-administered HIV testing in community-based settings

The early diagnosis and treatment of HIV infection has major economic implications for the health care system in terms of preventable infections and hospital admissions [41, 42]. Increased access to treatment has also been demonstrated as an effective approach to HIV transmission prevention among drug users [43, 44]. Through the PROUD study, we were able to offer HIV testing and referrals to our participants, many of whom may never have been identified through available clinical services.

Ottawa Public Health is at the forefront of HIV prevention for people who use drugs and leads practice and policy development for the city. The development of community-based peer-administered HIV testing stands to have a major impact on testing and treatment policies in the city and improve access to testing and treatment for people who use drugs.

Furthermore, the involvement of peers has implications beyond the moment of testing. Research has demonstrated that peers continue outreach efforts even when not compensated or supervised and reach people who use drugs in times and locations where risk behaviours are most likely to occur [45]. People with lived experience play an important role in reaching so-called “hidden populations”, building trust, and decreasing HIV risk behaviours through effective HIV prevention information [8, 39]. The successful integration of peers into these outreach efforts and the wider availability of HIV point-of-care tests create opportunities to further explore the role of peers in HIV testing and prevention efforts. The successful provision of peer-based testing through the current study demonstrates the feasibility of this approach. In order to optimally reach people who use drugs with HIV testing services, evidence supports the expansion of novel community-based methods for administering HIV POC tests, including peer-to-peer approaches.

### Conclusion and policy implications

The PROUD Study meaningfully engaged the communities of people who use drugs in Ottawa through the formation of the CAC, the training of peers as community-based researchers, and integrated KTE throughout the research project. This project successfully supported skill development across the team and empowered people with drug use experience to take on leadership roles, ensuring that this research process will promote change at the local level. The CBPR methods developed in this study provide important insights for future research projects with people who use drugs in other settings, and contribute to new knowledge of successful approaches to CBPR which take into account the meaningful engagement and self-determination of people most affected by HIV.
